# Additive effect of tDCS combined with Peripheral Electrical Stimulation to an exercise program in pain control in knee osteoarthritis: study protocol for a randomized controlled trial

**DOI:** 10.1186/s13063-017-2332-6

**Published:** 2017-12-21

**Authors:** Cleber Luz-Santos, Janine Ribeiro Camatti, Alaí Barbosa Paixão, Katia Nunes Sá, Pedro Montoya, Michael Lee, Abrahão Fontes Baptista

**Affiliations:** 1 Functional Electrostimulation Laboratory, Health Sciences Institute, Federal University of Bahia, Salvador, Brazil; 2 Graduate Program in Medicine and Health, Faculty of Medicine, Federal University of Bahia, Salvador, Brazil; 3 Bahian School of Medicine and Public Health, Salvador, Brazil; 4 Research Institute on Health Sciences, University of Balearic Islands, Palma de Majorca, Spain; 5 Graduate School of Health, Discipline of Physiotherapy, University of Technology Sydney, Sydney, NSW Australia; 6 Center for Mathematics, Computation and Cognition, Federal University of ABC, São Bernardo do Campo, São Paulo 09.080-045 Brazil; 7 Graduate Program in Neuroscience and Cognition, Federal University of ABC, São Bernardo do Campo, Brazil

**Keywords:** Knee osteoarthritis, Pain, Exercise, Transcranial direct current stimulation

## Abstract

**Background:**

Knee osteoarthritis (OA) has been linked to maladaptive plasticity in the brain, which may contribute to chronic pain. Neuromodulatory approaches, such as Transcranial Direct Current Stimulation (tDCS) and Peripheral Electrical Stimulation (PES), have been used therapeutically to counteract brain maladaptive plasticity. However, it is currently unclear whether these neuromodulatory techniques enhance the benefits of exercise when administered together. Therefore, this protocol aims to investigate whether the addition of tDCS combined or not with PES enhances the effects of a land-based strengthening exercise program in patients with knee OA.

**Methods:**

Patients with knee OA (*n* = 80) will undertake a structured exercise program for five consecutive days. In addition, they will be randomized into four subgroups receiving either active anodal tDCS and sham PES (group 1; *n* = 20), sham tDCS and active PES (group 2, *n* = 20), sham tDCS and PES (group 3, *n* = 20), or active tDCS and PES (group 4, *n* = 20) for 20 min/day for five consecutive days just prior to commencement of the exercise program. The primary outcomes will be subjective pain intensity (VAS) and related function (WOMAC). Secondary outcomes will include quality of life (SF-36), anxiety and depression symptoms (HAD), self-perception of improvement, pressure pain thresholds over the knee, quadriceps strength, and quadriceps electromyographic activity during maximum knee extension voluntary contraction. We will also investigate cortical excitability using transcranial magnetic stimulation. Outcome measures will be assessed at baseline, 1 month after, before any intervention, after 5 days of intervention, and at 1 month post exercise intervention.

**Discussion:**

The motor cortex becomes less responsive in knee OA because of poorly adapted plastic changes, which can impede exercise therapy benefits. Adding tDCS and/or PES may help to counteract those maladaptive plastic changes and improve the benefits of exercises, and the combination of both neuromodulatory techniques must have a higher magnitude of effect. Trial registration: Brazilian Registry on Clinical Trials (ReBEC) – Effects of electrical stimulation over the skull and tight together with exercises for knee OA; protocol number RBR-9D7C7B.

**Trial registration:**

ID: RBR-9D7C7B. Registered on 29 February 2016.

**Electronic supplementary material:**

The online version of this article (doi:10.1186/s13063-017-2332-6) contains supplementary material, which is available to authorized users.

## Background

Osteoarthritis (OA) is a chronic degenerative disease, primarily affecting the articular cartilage and subchondral bone of a synovial joint [1]. Radiographic features of OA include degradation of the articular cartilage, subchondral sclerosis and osteophyte formation [2, 3]. OA primarily affects the large, weight-bearing joints such as the knee and hip [4, 5]. The hallmark symptom of OA is chronic joint pain, which contributes to functional limitation and is a major cause of reduced quality of life. OA is the leading cause of disability affecting up to 15% of the global population, that equates to approximately 630 million people worldwide [6]. In South America, the prevalence of arthritis and rheumatism has been reported to range between 23.8 and 56.0% [7]. In Brazil, direct OA data is not available but chronic knee pain affects approximately 11.2% of the population [8].

Knee OA affects around 10% of people over 55 years old, a quarter of whom are severely disabled [2]. Thus, knee OA poses a considerable economic burden to the community [9] due to indirect expenses incurred by patients, such as home adaptations, medications [10, 11], costs incurred for loss of employment and productivity [12]. A recent study in the United States revealed that lifetime direct medical costs of people with knee OA totaled US$129,600 [13]. Therefore, knee OA is considered a significant global public health problem, especially with an ageing population.

**Fig. 1:**
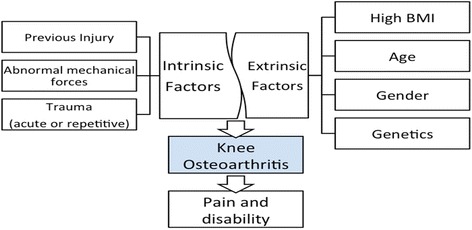
Multifactorial features of knee osteoarthritis composed of intrinsic and extrinsic factors

The development of OA is multifactorial (Fig. 1) but some main risk factors have been identified, and these include obesity, female gender, and previous knee injury [14]. However, previous research also noted the influence of age, genetic susceptibility, trauma (acute or repetitive), muscle weakness, joint laxity, and abnormal mechanical forces, such as repetitive kneeling and squatting, as important risk factors [15].

Abnormal mechanical stresses can impede natural repair and remodeling processes of the articular cartilage [16, 17]. The source of abnormal mechanical stress is diverse but has been associated with decreased postural control [18], muscle weakness and increased co-activation [19, 20], abnormal cumulative load [21, 22], joint instability [23], and the presence of abnormal tissue inside the joint (e.g., polymers of homogentisic acid oxidase in osteonecrosis) [16].

Conservative management, such as exercise therapy, designed to address issues like postural control and muscle dysfunction have limited success [24, 25]. Although the influence of OA on muscle strength is controversial, strengthening, stretching, and aerobic exercise are generally recommended [26]. A recent systematic review that pooled data from 44 trials concluded that land-based therapeutic exercises provide short-term pain relief (12 points/100) and improved physical function (10 points/100) for people with knee OA [27, 28]. After around 6 months, the benefits of exercise generally plateau, and pain often persist [27, 29]. The refractoriness to exercises needs to be addressed, and many factors may contribute.

An alternative explanation for the presence of persistent pain in OA patients is maladaptive neuroplastic changes in the spinal cord and brain [30, 31]. For example, a recent magnetic resonance imaging (MRI) study has demonstrated that patients with knee OA showed decreased volume in the somatosensory, insular, and motor cortices bilaterally. Two other clinical pain conditions were assessed including chronic back pain and complex regional pain syndrome. These changes are more pronounced in patients with a long duration of chronic pain, suggesting a positive correlation between chronicity of pain and decreased gray matter density. Similar reduction in volume has also been demonstrated in subcortical areas including the caudate nucleus and hippocampus in patients with knee OA and reduced volumes in those regions compromise motor control and learning [32].

The results from these imaging studies suggest that chronic pain is associated with functional changes in the brain. Studies using transcranial magnetic stimulation (TMS) have also provided useful information regarding the nature of corticomotor plasticity in chronic pain [33]. For example, several previous studies have demonstrated that various pain conditions, such as low back pain and fibromyalgia, are associated with decreased amplitude of motor evoked potentials (MEPs) and intracortical inhibition (ICI). Similar changes were also reported in studies utilizing experimental muscle pain [34–36]. To date, only few studies have examined cortical plasticity in patients with knee OA. Hunt et al. (2011) [37] in a single case study demonstrated that MEPs of the rectus femoris were decreased in unilateral knee OA, but this change could be reversed after 8 weeks of muscle strengthening, and corresponded to pain decrease. Kittelson et al. (2014) [38] found a significant negative association between the resting motor threshold (RMT) and pain, but no differences in cortical excitability measures between controls and OA subjects. Tarragó et al. (2016) demonstrated in patients with knee OA an association between chronicity of pain and reduction in intracortical inhibition [39]. Taken together, these findings suggest a central component related to chronic pain secondary to knee OA mostly indicating changes in M1 excitability, either decreased MEPs or motor thresholds, and decreased intracortical inhibition mechanisms and suggesting dysfunction in the interneurons which contribute to intracortical inhibitory circuits. Given the high functional connectivity of the motor cortex to other brain areas known to be involved in the pain matrix [40], such as the prefrontal cortex and thalamus [41], it might reflect not only impaired motor cortical function but also suggest a dysfunction in network connectivity as a potential mechanisms mediating the development of chronic pain [42].

As eluded to earlier, there is a growing body of evidence that many chronic pain disorders, including OA are associated with changes in motor cortical function [42–44]. Therefore, it is reasonable to suggest that treatment modalities that specifically target motor cortical function might have therapeutic value. It is well known that both exercises and electrical stimulation can induce cortical plasticity [45, 46]. It is possible that treatment effect maybe enhanced if exercise is combined with electrical stimulation. This seems as a relevant option to reverse these changes, but they may be not sufficient to accomplish a long-term effect and interfere with maladaptive changes in the brain [47]. Associating exercises with electrical stimulation may be an option to enhance therapy efficacy. It has been shown previously that cortical excitability could be modulated both centrally using TMS and TES [48, 49] or peripherally using electrical stimulation [50, 51], or the combination of both [52]. A novel therapeutic technique that has the potential to ameliorate maladaptive neuroplasticity in knee OA is Transcranial Direct Current Stimulation (tDCS). tDCS is a simple, safe, and non-invasive technique, which involves the application of low-intensity direct electric current to the scalp [53]. tDCS is known to acutely alter excitability of the motor cortex [54–56]. Anodal stimulation of the primary motor cortex can enhance excitability by increasing the neuronal resting membrane potential, while cathodal stimulation decreases it [57]. Anodal stimulation can induce analgesic effects [58, 59], probably through the modulation of neuronal membrane channels resulting in local and distant plastic changes [60]. To date, tDCS has been tested in a number of pain states with low-to-moderate effect size with an averaged reduction in a Visual Analogue Score (VAS) pain score ranging between 0.18 and 0.35 [59, 61–64].

However, studies using computational modeling with a Finite Elements Model (FEM) have demonstrated low focality and dispersion of current density through the scalp during tDCS [65, 66] which may contribute at least in part to its reported low effect size on clinical outcomes. Using tDCS alone, without any other active intervention, may be another explanation for its low efficacy reported by previous studies [61]. One way to test the above hypotheses is to investigate to effects of combining tDCS with Peripheral Electrical Stimulation (PES). PES, similar to tDCS, PES can also transiently modulate motor cortical excitability in a bidirectional way. Sensory and nociceptive PES decrease M1 excitability, while motor PES increases M1 excitability [50]. Previous studies have investigated the effect of combining these two neuromodulatory techniques – Schabrun et al. (2013) [52] have demonstrated that when anodal tDCS is combined with motor PES (both excitatory techniques), it neutralizes the excitatory effect of each other, leading to no significant changes in M1 excitability. This phenomenon can be explained by metaplastic mechanisms (homeostatic plasticity), a phenomenon necessary to maintain physiological integrity of a biological system through the limitation extreme changes in functioning [50, 67]. However, the combination of an excitatory brain stimulation technique (anodal tDCS) with an inhibitory peripheral stimulation (sensory PES) produced a more focal modulation, as seen in Paired Associative Stimulation. In this technique, a TMS single pulse is associated with an electric pulse in the median nerve [68] to increase or decrease excitability depending on the intensity or interstimulus intervals [69, 70]. Anodal tDCS has been combined with sensory PES in chronic low back pain individuals, resulting in a more pronounced pain reduction, and M1 reorganization [71]. Taken these results together, we hypothesized that the combination of anodal tDCS with sensory PES may have additive effects to exercises in people with knee OA.

There is encouraging evidence to suggest that the combination of tDCS and sensory PES may have the potential to reduce pain. However, it is currently unknown whether this combined approach is effective in patients with knee OA. Furthermore, it is unclear whether neuromodulation can augment the beneficial effects of exercise in patients with knee OA. Therefore, the double-blind, randomized, parallel study model was selected for this hypothesis.

## Hypothesis

The addition of anodal tDCS and sensory PES to an exercise program will have a more pronounced effect on decreasing pain and improving function in people with knee OA compare to exercise alone. Specifically, our hypothesis is that this combination will promote a more robust decrease of pain magnitude and improvement in function and quality of life. We further hypothesize that these changes will be correlated with increased excitability of corticomotor and cortico-cortical connections in M1, and quadriceps strength, independent of the presence of anxiety and depression. We also hypothesize that participants submitted to this regimen will maintain the benefits of stimulation 1 month after the end of the intervention, which will be associated with a self-perception of improvement.

## Objectives

### Primary

The primary aim is to determine if the addition of tDCS and PES to an exercise program is more effective in reducing pain, improving function and quality of life in patients with chronic knee OA compared to exercise alone.

### Secondary

To assess the effect of combining tDCS, PES and exercise, on motor threshold, cortical excitability, and cortical silent period of the rectus femoris, vastus medialis, and vastus lateralis muscles to exercise alone.

To correlate the neurophysiological findings with parameters of pain, muscle strength, global function related to OA, anxiety and depression symptoms, quality of life, and treatment perception from subjects.

## Methods

### Target population and sample

Participants will be recruited from public announcements and interviewed at the Laboratory of Functional Electrical Stimulation, Federal University of Bahia, Salvador, Bahia, Brazil. Sample size was estimated based on an effect size of 0.50 on pain relief measured by VAS score, study power of 80%, and four groups, with a total of 15 subjects per group. Total sample size was increased to 20 to account for loss to follow-up.

### Inclusion criteria

Participants will be included in this study if they have OA of the knee based on the clinical and radiological criteria defined by the American College of Rheumatology Society. These criteria include:

1. Age older than 50 years, knee pain on most days of the past month, osteophytes on plain X-ray and pain or difficulty in rising from sitting or climbing stairs [72]
2. Have a Chronic Pain Grade (CPG) score equal to or greater than II [73, 74]

### Exclusion criteria

Participants will be excluded if they:

1. Have a contraindication for TMS use such as: existence of metal in the skull or implanted devices, epilepsy history, pregnancy, use of drugs that might affect cortical electrical activity (anticonvulsants, antidepressants or antipsychotics), and complications with exposure to magnetic fields (TMS or MRI)
2. Have a disease history that might interfere with the knee OA, becoming a confounding bias – fibromyalgia, systemic lupus erythematosus, fractures of the knee region, knee prosthesis, low back impairment – that cause symptoms in the knee and peripheral nerves
3. Are incapable of comprehend the content from the assessments tools

### Discontinuity criteria

Procedures will be discontinued if:

- Moderate-to-severe adverse events are present, even related to electrical stimulation, exercises, or electrophysiological assessment
- Participants who initiate any other medical intervention to treat knee OA or other that may interfere with the results of this study

Subjects who initiate any of the treatments described below will be discontinued from the study:

- Physiotherapeutic treatment (conventional physiotherapy, postural re-education, pilates)
- Psychiatric treatment (associated anxiety and depression)
- Inclusion of new drug treatment for pain due to knee OA
- Treatment for weight reduction for obese individuals (aerobic exercise program)
- Fitness muscle training

### Study design

**Fig. 2:**
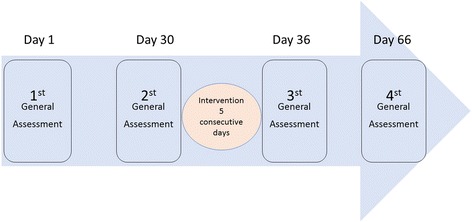
Participants’ assessment occurs in sequential moments: **a** Baseline assessment (day 1) – 1 month before the beginning of the intervention. **b** Revaluation 1 month later (day 30). **c** intervention for five consecutive days. **d** Reassessment at the conclusion of the exercise program (day 36) and 1 month after the last intervention day (day 66)

Study participants will be assessed on four different occasions: (1) baseline assessment (day 1) 1 month before the commencement of intervention; (2) reassessment a month later (day 30), (3) participants will be submitted to an intervention consisting of electrical stimulation applied before the structured exercise program for five consecutive days, (d) reassessment at the completion of the exercise program (day 36) and 1 month after the last day of intervention (day 66) (Fig. 2).

**Fig. 3:**
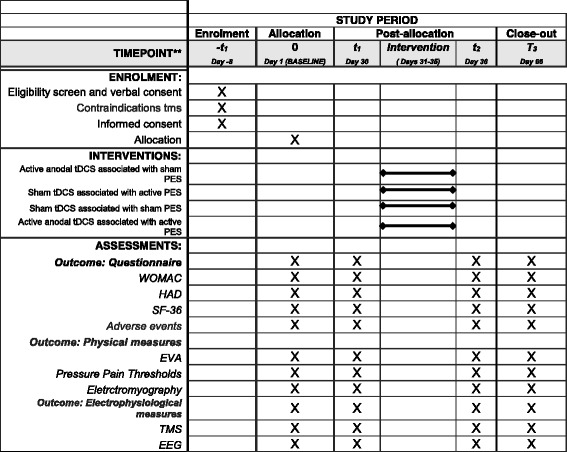
Schematic diagram of enrollment, interventions, assessments, and visits for participants

An experienced physiotherapist blinded to the study will perform initial neurophysiological assessment. The subjects will be randomly allocated by the research manager, which will only be involved in this study phase, at one of the four groups through a lottery system generated from a random-number table (www.randomization.com). Allocation sequence implementation mechanism will be by opaque and sealed envelopes. Access to this envelope will be exclusive to the physiotherapist who will apply the intervention techniques. The absence of blinding will not be allowed in this study. In the event of a breach of the blinding, the participant will be removed from the research protocol. The SPIRIT checklist with the registration events, interventions, evaluations and visits for participants can be verified in Fig. 3.

The same physiotherapist that performed the baseline assessment will teach and supervise the exercise program, common to all groups. Another researcher will be responsible for administering electrical stimulation interventions (tDCS, PES, and their combinations) and will not be involved in any other component of the study. The allocation concealment will be evaluated at the end of the intervention by custom questionnaire. The study participants will complete an adverse effect questionnaire at the end of the trial.

### Research groups

All groups will receive a supervised exercise program:

1. Active anodal tDCS associated with sham PES (*n* = 20)
2. Sham tDCS associated with active PES (*n* = 20)
3. Sham tDCS associated with sham PES (*n* = 20)
4. Active anodal tDCS associated with active PES (*n* = 20)

### Strategies to improve adhesion to the intervention

All the participants will receive compensations for transportation to and from the testing laboratory as well as a meal allowance. At the end of the study the intervention that promoted greater reduction in pain will be offered to those who did not receive it. To minimize withdrawal of participants during the study, a collaborating researcher will make telephone contact to confirm the appointment. Despite this, if the participant is absent, they may attend within 48 h for the evaluations without risk of exclusion from the protocol.

### Intervention description

#### Electrical stimulation

For the electrical stimulation protocol, participants will be comfortably seated on a chair at the Functional Electrical Stimulation Laboratory in the Health Science Institute of Federal University of Bahia. The procedures will be clearly explained and subjects will be encouraged to ask questions regarding the experimental procedures. The tDCS protocol will consist of a direct current stimulation lasting 20 min, of 2-mA amplitude, for five consecutive days before the exercise protocol, through a proper stimulator device (Soterix, New York, NY, USA). The anode will be placed on the primary motor cortex (M1 – C3 or C4, per the 10/20 international electroencephalogram (EEG) system) contralateral to the painful knee or the most symptomatic one in case of bilateral pain. The cathode will be placed on the opposite supraorbital region (Fp1 or Fp2, per 10/20 international EEG system). For the sham tDCS group the electrodes will be placed and the electric current connected for 30 s, then turned off and removed at the end of the 20-min period.

PES of the knee joint will be performed on its lateral and medial portions with a clinical pulse generator (Endophasys, KLD Medical Products, Brazil). Only the symptomatic side will be stimulated or the more symptomatic (in case of bilateral symptoms). A biphasic, symmetric current will be used for stimulation with a 0.25-ms pulse width, frequency of 10 Hz, with an amplitude at the sensory threshold, lasting 20 min, with the subject sitting comfortably at rest. Two surface electrodes of silicon-carbon bond, 5 × 5 cm, fixed on skin by Velcro will be used at each side of the joint. For sham stimulation the same electrode montage will be used with active stimulation on a sensory threshold for 30 s and then returned to zero. Electrodes will be removed after 20 min. tDCS and PES will start and stop at the same time during each session.

#### Exercise

The exercises will be based on a previously described protocol (Bennel and Dobson (2014) [75]), and will be taught by a trained physiotherapist at the first day of intervention. Assistance to proper performance and progression will happen during the next 4 days’ intervention. In summary, the protocol consists on strengthening exercises for muscles of the knees and hips. The intervention will be carried out with three series of 8 to 12 repetitions (Additional file 1).

## Outcomes

### Primary outcome measures

#### Pain

The intensity of the symptomatic knee (or the knee with greatest symptoms if both knees are affected) will be assessed before and after each intervention session. In case of equal magnitude of pain on both knees, the dominant side will be evaluated. Pain magnitude will be assessed at all meetings with the participants, through a 0–10 VAS. When this meeting includes the intervention, pain will be assessed before and after the procedures. In general evaluations, the pressure pain threshold on the medial and lateral sides of both knees will be assessed using a digital pressure pain threshold (EMG System, São José dos Campos, Brazil). Pressure pain threshold at the medial and lateral sides of both knees will be assessed using a digital pressure pain threshold meter (EMG System, São José dos Campos, Brazil) on days 1, 30, 36, and 66. The mean of three measures at each point will be used for analysis. Together, pain, stiffness and physical function will be assessed through the Brazilian version of the Western Ontario and McMaster Universities Osteoarthritis Index (WOMAC) [76].

### Secondary outcomes

The secondary outcomes will be assessed at each of the four general assessment sessions (days 1, 30, 36, and 66). Quality of life will be assessed through the 36-item Short Form (SF-36) questionnaire [77] and symptoms of depression and anxiety through the Hospital Anxiety and Depression Scale (HADS) [78].

Quadriceps muscle strength will be assessed through a digital dynamometer (EMG System, Brazil) positioned at a right angle to the ankle. The subject will be seated with hip and knees at 90°. The make test paradigm will be used. Three maximal isometric contractions of both knees will be recorded, each lasting 6 s, with 1 min rest between them. Standard verbal encouragement will be provided to all participants to generate maximal force. The average of the three recordings will be used for subsequent analysis.

Cortical excitability will be assessed using a Magstim BiStim stimulator (Magstim Co. Ltd, Dyfed, UK). The quadriceps area corresponding to the painful knee and the anterior superior iliac spine (ASIS) will be cleaned with alcohol. Self-adhesive electromyography (EMG) electrodes Ag/AgCl (Noraxon, Scottsdale, AZ, USA) will be positioned on the muscle belly of the rectus femoris, vastus lateralis and vastus medialis muscles. The RMS EMG activity will be pre-amplified x3000, filtered at 1–2000 Hz, and sampled at 4000 Hz using a 1401 acquisition system and Signal v.06 software (Cambridge Electronic Design, Cambridge, UK). During the testing procedure the subject should extend the knee until an EMG activity of 100 μV is reached. The participant will be asked to maintain this level of contraction during the entire evaluation period.

The vertex will be marked at the intersection of the *interaural* and *nasion* to *inion* lines according to the 10/20 EEG system. The subject will be comfortably seated on a proper chair and kept awake during the entire evaluation protocol. A polyester cap previously marked with a 1 × 1-cm grid will be positioned on the subject’s head and will serve as reference for the TMS. The TMS will be applied by the examiner using a figure-of-eight coil to deliver single and paired pulsed TMS to the motor cortex, while RMS EMG activity will be monitored in real time through Signal v.06 software (Cambridge Electronic Design, Cambridge, UK). The spot where a balanced EMG activity is found for the rectus femoris, vastus lateralis, and vastus medialis activation will be considered as the “hot spot.” Active motor threshold (AMT) will be estimated as the minimum stimulator intensity that evokes responses in the target muscle of at least 200 μV of peak-to-peak amplitude. AMT will be estimated through computer software (Motor Threshold Assessment Tool, www.clinicalresearcher.org). MEP amplitude will be estimated by the mean of 10 pulses at 120% of AMT. For short-interval intracortical inhibition (SICI) and intracortical facilitation (ICF), a subthreshold conditioning pulse (80% AMT) will be delivered followed by a suprathreshold test stimulus (120% AMT) separated by pre-defined intervals of 2 ms for SICI and 15 ms for ICF.

### Potential TMS risks

Potential risks were described by Rossi et al. (2009) and Lefaucheur et al. (2011) [79, 80]. All potential risks are listed and summarized on the subject’s Informed Consent Form. Subjects will complete an adverse effects questionnaire for TMS and tDCS just after each intervention.

### Ethical aspects

The protocol meets the legal requirements involving research in humans, according to the Declaration of Helsinki. All participants will be briefed on the objectives and will sign the Consent Form. This protocol was approved by the Ethics Committee of the Health Sciences Institute of the Federal University of Bahia under number 1,378,100, and in the Brazilian System of Clinical Trials Registration (REBEC) under number RBR-9D7C7B. Possible changes to the protocol will be communicated to the Ethics Committee, REBEC, and participants. The findings will be forwarded to the participants and collaborators through a simplified report in an accessible language. Documents will not be made public without the permission of the participant and the resulting publications will have no identification of the subjects. The data will be archived with the principal investigator for 5 years and then destroyed. Researchers are physiotherapists who are able to help in cases of research-related harms.

The authors of the publications will be considered the researchers who meet the following criteria:

1. Substantially contributing to the design and planning, or acquisition of data, or analysis and interpretation of data
2. Participate in article writing or critical intellectual review
3. Review and approve the final version to be published

The researchers declare no conflicts of interest with financial agents and access to data will be restricted to key researchers.

### Consent to publish

Written consent will be obtained from the participants for the publication of their individual data and accompanying images in this manuscript. The Consent Form will be archived by the researcher in charge and will be available for review by Editor-in-Chief. The images provided to illustrate the exercise program were granted by one of the collaborators through the term of assignment of the image use.

### Data analysis

All statistical procedures will be performed according to the principles of intention-to-treat. A linear mixed model will be used to identify the differences in pain intensity (VAS), PPT, and WOMAC scores between the groups with real stimulation (tDCS, PES and tDCS + PES) and sham interventions across the periods of assessment (factors intervention and time). Anxiety and depression will be analyzed as covariables. When necessary, post hoc comparisons will be performed using the Bonferroni correction for multiple comparisons. Analysis of the cumulative proportion of respondents with different cutoff points will be employed to analyze clinical analgesic response between groups according to Farrar et al. (2006) [81]. Correlation measurements will be performed between the clinical variables (VAS, PPT, and WOMAC scores and secondary variables (quality of life and TMS variables PEM, IIC and FIC). All data will be analyzed using software SPSS v.23.0. Statistical significance will be set as *p* < 0.05.

## Discussion

We adhere to the recommendations of SPIRIT (Standard Protocol: Recommendations for Interventional Testing) in the development and reporting of our protocol (Additional file 2). The exercises have demonstrated positive effects on pain and function in various chronic pain conditions [82]. The results obtained can be optimized when associated with non-invasive neuromodulatory techniques. The procedures with the most evidence are from tDCS [83–85] and PES [86, 87]. The combination with the largest effect size is not sufficiently elucidated in the scientific literature and ideal estimation parameters of the modalities are controversial. The present study used the parameters with the highest scientific evidence for the proposed clinical outcomes.

Exercise combined with neuromodulatory techniques may reverberate in the motor cortex [46, 70]. This fact suggests that clinical and electrophysiological variables are positively correlate. Reorganization of the quadriceps motor cortex after an exercise program supports this correlation [70].

Clinical impacts may represent significant changes in the cortical activity of the subject with knee OA. The understanding of the relationship between brain and muscle may lead the physician to propose interventions that amplify this connection and significantly improve function.

### Benefits

All individuals will receive specific follow-up from physiotherapists, who will guide the implementation and evolution of therapeutic exercises with a good level of evidence for the treatment of OA of the knee. Additionally we believe that the electrical resources tested can increase the effects of the exercises. If these resources prove to be effective, the best therapy will be offered to those who did not receive it.

### Trials status

Estimated enrollment is 15 participants. The study start date was June 2016, and the estimated study completion date is June 2018.

## Additional files

Additional file 1: Description of the exercise program with images, progressions and repetitions. (PDF 1335 kb)

Additional file 2: SPIRIT 2013 Checklist: recommended items to address in a clinical trial protocol and related documents*. (DOCX 49 kb)
