# Supplementary material for: Additive effect of tDCS combined with Peripheral Electrical Stimulation to an exercise program in pain control in knee osteoarthritis: study protocol for a randomized controlled trial
Source: Trials. 2017 Dec 21;18:609. doi: 10.1186/s13063-017-2332-6 (PMC5740917; doi:10.1186/s13063-017-2332-6)
Supplement: Supplementary file 1 — Description of the exercise program with images, progressions and repetitions. (PDF 1335 kb) [file 13063_2017_2332_MOESM1_ESM.pdf]

Supplement 1 (or call this "Appendix"?)

EXERCISE PROTOCOL

Description of the exercise program with images, progressions and repetitions.

Exercise	Image	Progression	Repetition
**1. Strengthening of knee extensors**<br>Knee extensions in the sitting position. Proceed to use the weight of the other lower limb. In the sitting position, slowly stretch the symptomatic knee to the straightest position possible. Hold for five seconds and lower slowly.	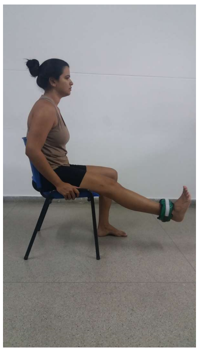	Use the weight of the other leg and then dock with the other leg against the knee extension.	3 sets of 10. 30 seconds interval between sets.
**2. Strengthening of hip abductors**<br>Level 1:<br>In lateral decubitus, abduction of the hip. Keep the body straight and knee straight. Do not take the lower limb forward. Keep the heel higher than your fingers and behind the hip line while bringing the lower limb to the ceiling. Hold for five seconds and then lower slowly.	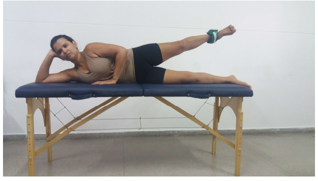	Increase the weight with shin guards or progress to level 2.	3 sets of 10. 30 seconds interval between sets.
Level 2:<br>Abduction of the standing hip, using elastic bands as resistance. Place a circular elastic band around both legs, just above the ankle. Maintain adequate tension in the elastic band and correct straight posture, positioning shoulders and hips facing forward before beginning the exercises. The backrest of a chair or the wall can be used for support. Hold for five seconds and then lower slowly.	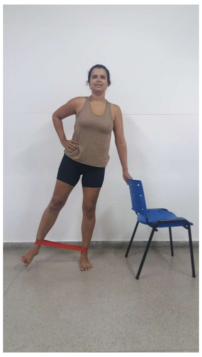	Increase the tension of the elastic bands.	3 sets of 10.<br>30 seconds interval between sets.

Exercise	Image	Progression	Repetition
**3. Discharge of knee weight / strengthening of hip extensors**<br>Level 1:<br>Partial squats on the wall (the image option includes adding an elastic band around the knees to include the abductor muscles of the hip).<br>Keep one foot 30 cm away from the wall, with separate feet and rotated internally.<br>With your back straight and your torso and buttocks leaning against the wall, slowly lower your body, sliding over the wall (as if you were sitting) until about 60o (less if painful) knee flexion and raise again. Keep in touch with the wall all the time.<br>The knees should be kept behind the toes during the squats.<br>Hold position for five seconds.	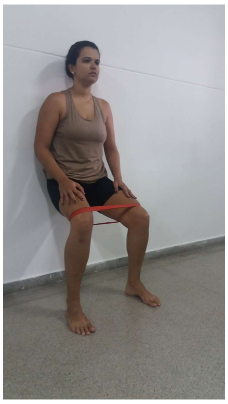	Increase the resistance by increasing the tension of the elastic bands.<br>Proceed to level 2.	3 sets of 10.<br>30 seconds interval between sets.
Level 2:<br><br>Sit-and-lift (option of adding elastic bands around the knees to incorporate the abductor muscles of the hip).<br>Sitting with your back resting on a medium height seat chair and firm seat, stand up slowly without using your hands for support.<br>Flemish the trunk forward so that the glutes are raised and the hips are below the trunk.<br>Hold the buttocks for 3 seconds discreetly outside the chair before sitting down slowly.	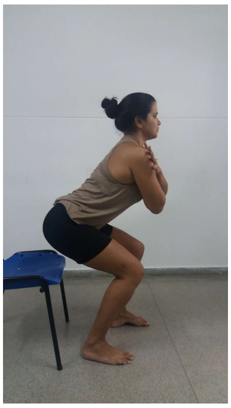	Increase the resistance by increasing the tension of the elastic bands.<br>Proceed to level 3.	3 sets of 10.<br>30 seconds interval between sets.
**4. Sitting-ischial-tibial strengthening**.<br>Put a circular elastic band around the leg and leaning against a heavy table or chair.<br>Sitting in a chair, place the symptomatic leg with the knee slightly bent. Slowly pull the leg back until the knee is more bent and a strong resistance is felt on the elastic band. Hold for 5 seconds.	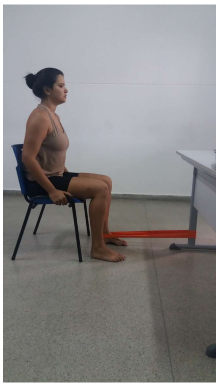	Increase squat depth	3 sets of 10.<br>30 seconds interval between sets.

Exercise	Image	Progression	Repetition
5. Steps:<br>a) Climbing steps:<br>Place the symptomatic leg on the step. Slowly climb the step. Move the foot from the non-symptomatic side to the step and then return with both feet in the initial position, on the floor.	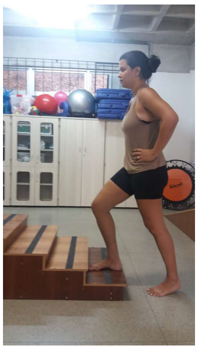	First increase the height of the step and then add weights. The weights should be held in the chest by both hands or two weights on the hands may be used.	3 sets of 10. 30-60 seconds of interval between sets.
b) Go down steps:<br>Start with both legs standing on the step.<br>Bend the knee on the affected side slowly, to bring the unaffected side toward the ground. Then stretch the affected knee to slowly return to the starting position.<br>The knee on the affected side should point forward during movement.	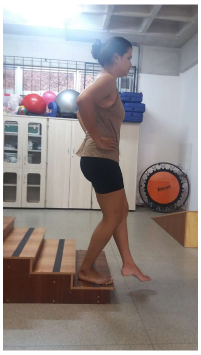	First increase the height of the step and then add weights. The weights should be held in the chest by both hands or two weights on the hands may be used	3 sets of 10. 30-60 seconds interval between sets<br>.
